# Prevalence of heroin and methamphetamine male users in the northern Taiwan, 1999–2002: capture-recapture estimates

**DOI:** 10.1186/1471-2458-7-292

**Published:** 2007-10-17

**Authors:** Shu-Chuan Chiang, Chuan-Yu Chen, Yuan-Ying Chang, Hsiao-Ju Sun, Wei J Chen

**Affiliations:** 1 Taoyuan Mental Hospital, Executive Yuan-Department of Health, 71 Longshou St., Taoyuan City, Taoyuan County 330, Taiwan; 2 Ching Yun University, 229 Chien-Hsin Road, Jung-Li 320, Taiwan; 3 Division of Mental Health and Substance Abuse Research, National Health Research Institutes, 390 Sung-Te Rd., Taipei 110, Taiwan; 4 Department of Emergence Medicine, Tao-Yuan Veterans Hospital, 100 Cheng – Kung Road Sec. 3, Tao – Yuan 330, Taiwan; 5 Institute of Epidemiology, College of Public Health, National Taiwan University, 17 Xu-Zhou Road, Taipei 100, Taiwan; 6 Department of Psychiatry, College of Medicine and National Taiwan University Hospital, National Taiwan University, 7 Chung-Shan South Road, Taipei 100, Taiwan

## Abstract

**Background:**

Illegal drug use and related problems have been emerging as an important public health issue in Taiwan. Via the capture-recapture approach, the present study aimed to offer insights into the size of heroin and methamphetamine male user population in the northern Taiwan during the period from 1999 to 2002.

**Methods:**

Annual lists of male subjects were collated from both judiciary and medical systems in Taoyuan County, Taiwan. A total of 2809, 2486, 1661, and 1440 local male illegal drug users aged 15 to 54 years were identified in Taoyuan County from 1999 to 2002, respectively.

**Results:**

An estimated number of 16192, 14532, 16844, and 11783 local male methamphetamine or heroin users were found in each of the four consecutive years in the region. From 1999 to 2002, the annual prevalence rate for heroin use was 0.27% (95% CI = 0.20%, 0.38%), 0.33% (95% CI = 0.25%, 0.44%), 0.63% (95% CI = 0.44%, 0.92%), and 0.72% (95% CI = 0.54%, 0.97%), respectively, suggesting a trend of significant increase (chi-square for linear trend = 1677.76, d.f. = 3, p < 0.0001). In contrast, a decreasing trend was found for methamphetamine (2.38%, 1.91%, 2.47%, and 1.24%), with a modest rebound in 2001. The prevalence rates of illegal drug use for male residents in Taoyuan County were approximately 2–3% during this period, and the scale of problem shows no sign of diminution.

**Conclusion:**

By taking advantage of existing datasets that were incomplete by each alone, the approach of capture-recapture model may be ultimately considered as a tool to estimate the scale of illegal drug use problems. The population of heroin-using males apparently is stably expanding in the northern part of Taiwan in the first years of 21^st ^century.

## Background

Illegal drug use and associated problems have emerged as an important public health problem in many parts of the world [1-3]. The involvement of illegal drugs is associated with not only increased occurrence of other psychiatric disorders (e.g., psychosis), but also elevated risk of blood-borne infectious diseases, such as hepatitis C virus (HCV) and human immunodeficiency virus (HIV)/acquired immunodeficiency syndrome (AIDS). Moreover, although the causality is still debated, illegal drug use was commonly found connected with criminal acts that pose general threat to public safety and security in a notable degree. Up to date, a number of developed countries have invested enormously in establishing monitoring systems to investigate the size of illegal drug use problems and related consequences over time [1,4-8], which is of great value for assessing the need for treatment, devising evidence-based clinical protocols, planning the distribution and delivery of health care, and developing preventive programs associated with addiction [5,6]. Notwithstanding these remarkable advantages, such systems generally remain impractical in the countries wherein resources are limitedly available or other health problems take a heavier toll.

Because use of illegal drugs is subject to criminal prosecution in Taiwan, population-based surveys or reporting systems are usually vulnerable to the methodological limitations such as under reporting, differential ascertainment process, and incomplete representativeness. To reduce the limitations inherited in different datasets, investigators have proposed several approaches to obtain relatively valid and precise estimates of prevalence rate by utilizing multiple incomplete systems, among which the capture-recapture method is commonly used [9-11]. The rationale is that by re-weighting the overlapped proportion from 'captured' and 'recaptured' samples between different source systems, we can indirectly estimate the size of the undetected. Strict assumptions in earlier approaches of capture-recapture models have limited application in research involving human populations [9,10]. Recently, with the introduction of more flexible methods to deal with multiple systems, epidemiologists have paid increasing attention in adopting this approach to investigate disease distribution in the general population [9-12]. Even so, to this point, evidence derived from capture-recapture models on the topic of illegal drug use in the general population was still scant [13-17].

According to the official judiciary statistics of arrestees [18] and addiction services provided by psychiatric hospitals [19], methamphetamine and heroin were two of the most commonly consumed illegal drugs in Taiwan. However, there have been few epidemiological studies investigating the prevalence of illegal drug use in the general population, and most of the study samples were limited to young populations [7,20-22]. With respect to adult population, one survey among community subjects found that 0.8% of females and 1.3% of males had ever tried illegal drug once in their lifetimes [23]. In terms of substance use disorders, the reported prevalence was even lower; in one case only 0.16% of drug abuse/dependence was found in a community survey conducted in 1982–1986 [24,25]. In general, these studies relied primarily on self-report and, therefore, were likely to be underestimated [26].

Against this backdrop of possible limitations in self-report surveys, we then turn to the approach of capture-recapture model. By utilizing the available datasets from two different sources – medical and judiciary systems, we sought to offer insights into the size of heroin and methamphetamine use problems and its possible changes recently observed in Taiwan communities. The aims of the present study were to estimate the numbers of heroin and methamphetamine-using males in northern Taiwan over time during the period from 1999 to 2002, and to examine the prevalence rates of heroin and methamphetamine use in Taoyuan County over the same study period.

## Methods

### Source of data

Data were obtained from two different recruitment processes: one is from judiciary system (i.e., Taoyuan Prison), and the other is from medical system (i.e., Taoyuan Mental Hospital, Taoyuan Veterans Hospital, and Ju-Shan Psychiatric Hospital). Taoyuan Prison is the only male prison in Taoyuan County, which takes charge of detoxification for all arrested male users of illegal drugs. Taoyuan Mental Hospital (the designated center for addiction treatment in northern Taiwan), Taoyuan Veterans Hospital, and Ju-Shan Psychiatric Hospital, which were the largest three psychiatric centers in Taoyuan County, have more than seventy psychiatric beds separately and provide outpatient and inpatient treatment for drug-related disorders. In order to estimate the overlapping and non-overlapping numbers of drug users across judiciary and medical systems, personal identification number was used to collate the data from two recruitment sources. Among these subjects, only local residents aged 15–54 years were taken into account while estimating population size and prevalence rates of drug use in Taoyuan County. Annual statistics of male residents aged 15–54 years were 532378, 547664, 558908, and 570034 through 1999 to 2002 in Taoyuan County, respectively [27].

According to Taiwan's laws, any male illegal drug user who is arrested within Taoyuan County and has positive urinalysis for any illegal drugs is adjudicated for detoxification in Taoyuan Prison. The majority of illegal drug users were involved in the use of either methamphetamine or heroin. Individuals on the list of detoxification-unit in Taoyuan Prison from 1999 to 2002 were included for this study. The list contained compulsory items, such as subject's name and a designated number, as well as optional ones, such as personal identification number, birthday, residential status, and the type of illegal drugs involved. The proportion of individuals with any optional item missing was about 35% in 1999, 10% in 2000 and 2001, and less than 3% for 2002. For those subjects with missing information, additional effort was made to match name, designated number, and an array of optional items on the list with the dataset at the Taoyuan Prison, which had personal identification number, birthday, and residential status.

With respect to the hospital source, patients with illegal drug-using history were recruited on the basis of clinical diagnostic codes. All male patients who met either methamphetamine- or heroin-associated diagnosis (such as abuse, dependence, or associated psychosis) during the period of 1999–2002 were included. All the three participating hospitals adopted the diagnostic system of the International Classification of Diseases-10 (ICD-10) [28], and the first author, a board certified psychiatrist with the specialty in drug abuse treatment, performed chart review to confirm the diagnosis for any unspecified substance-associated disorder found in the computerized data system.

Any case who was involved in the use of methamphetamine, such as having methamphetamine-use crime or methamphetamine-associated diagnosis, was coded as a methamphetamine user, and the same procedure was applied to identify heroin users. The interchanging or polydrug users (interchanging or combined use of methamphetamine and heroin) were coded as users of both methamphetamine and heroin in our analyses.

Access to the personal identification and relevant information of all study subjects was granted permission by both the Ministry of Justice and the Taoyuan Prison, as well as by the institutional review boards of the three participating hospitals.

### Statistical analysis

In this study, the prison cases were considered as a random sample representative of illegal drug users in the community since they were ascertained via the practice of law enforcement. On the other hand, hospital cases represented a highly selective subgroup of illegal drug users with severe forms of clinical problems and hence in need of intensive help from medical professionals. Thus, we assumed intuitively that the subjects recruited from medical system were sampled from a sub-population of the general illegal drug-using population – the same source population for the subjects recruited from judiciary system. Given the assumption of one randomized sample, the local dependence or correlation between the two lists was less likely to exist and hence rendered the capture-recapture-derived estimates unbiased [11]. The undetected size *n *is estimated as *bc/a*, where *a *is the size of overlapping cases, and *b *and *c *are the sizes of non-overlapping cases in either systems, respectively. The 95% CI of estimated target (*a*+*b*+*c*+*n*) was calculated using log-transformations, i.e., treating log(n) as an approximately normal random variable [29]. Then the prevalence rate is estimated by (*a*+*b*+*c*+*n*)/*N*, where *N *is the size of resident population, and its variance as (Variance of *n*)/*N*^2^. The trend of prevalence was examined by Cochran-Mantel-Haenszel (CMH) tests. All analyses were performed using computer package program SAS [30]. A *P*-value of less than 0.05 was considered significant.

## Results

The numbers of illegal drug users ascertained during the study period of 1999 to 2002 are shown in Table 1. In terms of sample size, the number of arrestees for illegal drug use was found reduced from 1999 to 2002. In contrast, for hospital-recruited cases, the number of methamphetamine users was steady and that of heroin users increased over the year. Comparing the ages of cases, those of hospital-recruited cases tended to be slightly older than their prison-recruited counterparts, particularly for methamphetamine users. In general, the mean age of heroin users was two years older than that of methamphetamine users. About 80% to 91% of ascertained cases were local residents (i.e., Taoyuan County).

**Table 1:** Selected characteristics of ascertained methamphetamine and heroin male users aged 15–54 years, by year and source, Taoyuan County, Taiwan

		Total		Resident only^a^		
				
Year	Drug	N	Age Mean (SD)	N	Age Mean (SD)	Resident/total %
Prison						
1999	All^b^	3126	28.6 (7.8)	2547	28.5 (7.7)	81.5
	MeA^c^	2860	28.4 (7.8)	2327	28.2 (7.8)	81.4
	Heroin^d^	399	31.2 (6.6)	330	31.0 (6.6)	82.7
2000	All	2542	28.7 (7.8)	2188	28.5 (7.7)	86.1
	MeA	2184	28.2 (7.8)	1865	28.0 (7.8)	85.4
	Heroin	372	31.5 (7.2)	337	31.1 (7.1)	90.6
2001	All	1483	30.1 (8.0)	1299	29.9 (7.9)	87.6
	MeA	1095	29.3 (8.0)	956	29.1 (7.9)	87.3
	Heroin	393	32.4 (7.7)	347	32.2 (7.6)	88.3
2002	All	1012	30.9 (7.9)	867	31.0 (7.9)	85.7
	MeA	624	30.0 (7.8)	532	30.2 (7.8)	85.3
	Heroin	400	32.4 (7.7)	347	32.3 (7.8)	86.8
Hospital						
1999	All	322	30.1 (6.9)	267	30.0 (7.0)	82.9
	MeA	215	29.2 (6.60	180	29.2 (6.8)	83.7
	Heroin	124	31.6 (7.1)	104	31.3 (7.0)	83.9
2000	All	404	30.8 (7.3)	352	30.7 (7.5)	87.1
	MeA	202	29.8 (6.7)	174	29.9 (6.9)	86.1
	Heroin	216	31.5 (7.8)	192	31.3 (7.9)	88.9
2001	All	460	32.3 (7.3)	389	32.5 (7.4)	84.6
	MeA	198	30.9 (6.7)	159	31.3 (6.8)	80.3
	Heroin	265	33.4 (7.7)	233	33.3 (7.7)	87.9
2002	All	702	33.2 (7.5)	598	33.4 (7.5)	85.2
	MeA	239	31.8 (6.8)	212	31.9 (6.7)	88.7
	Heroin	507	33.7 (7.7)	424	33.9 (7.7)	83.6

^a^Individuals with documented addresses located in Taoyuan County, Taiwan.

^b^Any case who was involved in the use of either methamphetamine or heroin.

^c^Any case who was involved in the use of methamphetamine.

^d^Any case who was involved in the use of heroin.

**Figure 1:**
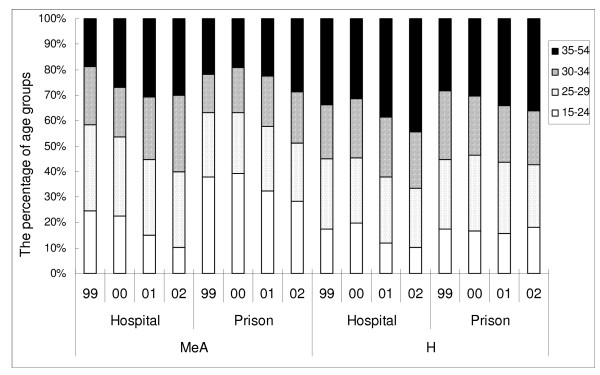
Age distribution of illegal drug male users in Taoyuan County, Taiwan, by source, drug type, and year.

To better illustrate the age composition of these illegal drug users, we divided age into four intervals and the corresponding percentages are displayed in Figure 1. For methamphetamine users, regardless of sources, the percentage of the youngest group (15–24) decreased whereas that of the oldest group (35–54) increased over the years. For heroin users, the percentage of the oldest group was the greatest one of the four age groups for hospital-recruited cases, and the percentage continued to expand over the year. In contrast, the age distribution remained stable for prison-recruited heroin cases.

On the basis of male subjects aged 15–54 years old, capture-recapture analyses were carried out to estimate the size of heroin and methamphetamine users over four study years. As shown in Table 2, the largest estimate of methamphetamine users was found in 2001, and the corresponding estimate for heroin was in 2002. The estimates of heroin users were increased steadily from year 1999 to 2002. On the contrary, a decreased trend was found for methamphetamine, with a modest rebound in 2001.

**Table 2:** Estimated numbers and prevalence of illegal drug users by year, Taoyuan County, Taiwan

	1999			2000			2001			2002		
	
Variable	All^a^	MeA^b^	H^c^	All	MeA	H	All	MeA	H	All	MeA	H
Captured users, resident of Taoyuan County												
Prison only	2505	2294	306	2135	1834	301	1269	945	324	823	516	311
Hospital only	225	147	80	299	143	156	359	148	210	554	196	388
Overlap	42	33	24	53	31	36	30	11	23	44	16	36
Estimate of undetected users	13420	10219	1020	12045	8460	1304	15186	12715	2958	10362	6321	3352
Estimate of total users	16192	12693	1430	14532	10468	1797	16844	13819	3515	11783	7049	4087
Lower bound of 95% C.I.^d^	12421	9485	1049	11463	7749	1385	12111	8071	2462	8998	4528	3079
Upper bound of 95% C.I.	21436	17368	2037	18649	14475	2400	23719	24309	5150	15593	11241	5528
Estimate of the prevalence^e^	3.04%	2.38%	0.27%	2.65%	1.91%	0.33%	3.01%	2.47%	0.63%	2.07%	1.24%	0.72%
Lower bound of 95% C.I.	2.33%	1.78%	0.20%	2.09%	1.41%	0.25%	2.17%	1.44%	0.44%	1.58%	0.79%	0.54%
Upper bound of 95% C.I.	4.03%	3.26%	0.38%	3.41%	2.64%	0.44%	4.24%	4.35%	0.92%	2.74%	1.97%	0.97%

^a^Any case who was involved in the use of either methamphetamine or heroin.

^b^Any case who was involved in the use of methamphetamine.

^c^Any case who was involved in the use of heroin.

^d^Confidence interval by using log-transformation

^e^During the period 1999–2002, the annual statistics of male residents aged 15–54 in Taoyuan County were 532378, 547664, 558908, and 570034, respectively.

**Figure 2:**
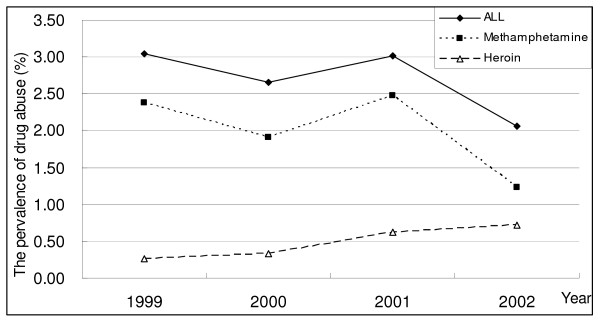
Estimated prevalence of illegal drug male users by drug type and year, Taoyuan County, Taiwan.

As shown in Figure 2, the prevalence of heroin-using males in study population was stably increasing over the four years of the study (chi-square for linear trend = 1677.76, d.f. = 3, p < 0.0001), and roughly three in four hundred males in Taoyuan County had used heroin once in year of 2002. In contrast, the prevalence of methamphetamine use seems decreasing in the same period, and an estimated 2% of males have used either methamphetamine or heroin at least once in 2002.

## Discussion

By taking advantage of two existing datasets of which each was incomplete, our results demonstrate that the approach of capture-recapture method may be helpful in providing the empirical evidence with respect to the subpopulation of illegal drug users in the community. During the period of 1999–2002, the prevalence rates of illegal drug use for males residing in Taoyuan County were approximately 2–3%, and the scale of problem shows no sign of diminution. These prevalence estimates are much higher than those obtained from prior studies conducted in Taiwan [24,25]. Some issues pertaining to the application of the capture-recapture method in the investigation of illegal drug use problems would be discussed as followed.

### Characteristics and population size and of illegal drug users

The ages for the captured illegal drug users in either judicial or hospital system peaked roughly from late twenties to early thirties. Regardless of source, the mean age of illegal drug users in 2001 and 2002 is slightly older than that of 1999 and 2000. Two explanations might, in part, account for the observed differences in relation to age. First, the majority of illegal drug users in 2002 might be the same group ascertained from previous years, with unremitted or recurrent illegal drug-using problems. Second, there were really new but older users who just had their first illegal drug in 2002, such as later-onset users of heroin. Given the observed age-related changes over time, some implications for prevention and treatment may be considered. For example, age- or development-tailored strategies should be devised to target initiation of illegal drug in adulthood. It is very possible that these late-onset heroin or methamphetamine users may exhibit the profile of risk factors different from that of adolescent-onset illegal drug users. In addition, effective treatments and follow-up on the issue of chronicity of addictive disorders may be as important as primary prevention in adolescents. It is noteworthy that, in our study samples, methamphetamine users were generally younger than their corresponding heroin-using counterparts, indicating that methamphetamine in Taiwan may serve as a transitional role in the process leading to the most advanced stage of illegal drug use (i.e., heroin). From the perspective of prevention, these results may suggest that more programs are needed to treat younger-age drug users involved in less advanced illegal drugs (e.g., methamphetamine), in order to reduce the subsequent progression into the most advanced illegal drug in their later lives [31]. In this study, the proportion of combined methamphetamine and heroin users was small (2% for the prison source and 5% for the hospital source). Throughout the four study years, the mean age of combined drug users from prison (30.0 ± 6.9, n = 140) was close to that from hospital (30.0 ± 6.8, n = 73). The clinical characters of these combined drug user are not well known yet and need further investigation.

The estimated number of arrested drug users was found gradually lowered over the study period. The diminution of arrested illegal drug users might be, in part, explained by a shift in the main tasks executed by law enforcement with time in Taoyuan County (e.g., from illegal drugs to other criminal activities). The reduced number of methamphetamine users recruited from judiciary system could also be attributed to relatively easy and wide availability of other illegal drugs during the same period in Taiwan (such as ecstasy, ketamine, or others) [32]. These emerging categories of illegal drugs may have replaced or shared the role of methamphetamine in illegal drug market. Moreover, according to the regulation of the new drug's law (the Statute for Narcotics Hazard Control) valid since 1998, the duration of detention is influenced by accumulated times for recidivism on illegal drugs or the risk for recurrence assessed during the detoxification period [33]. Most of the second-time arrestees for illegal drug use will usually stay for a longer period of detention in order to maintain abstinence (e.g., 5–12 months). Those who have recommitted illegal drugs use for three times or more will be imprisoned in jails for even longer. Since it is very unlikely for those who were imprisoned or were kept in prolonged detention to be arrested during the serving period, the observed diminution in the population of arrested illegal drug users might be a result of the initial enactment of the New Drug Act since 1998.

Given that illegal drug-using individuals who sought medical help or were arrested elsewhere are included in neither of two datasets, the prevalence rates obtained in the present study are likely to be underestimated. Even so, the estimate on prevalence of male illegal drug use (2.07%–3.04%) in Taoyuan County is still much higher than that of previous studies either by psychiatric diagnosis (<1%) [24,25], or lifetime use experience (1.3%) [23]. It is interesting to note that a recent surveillance among patients at emergency departments in Taiwan revealed that the prevalence of any illegal drug use by self-report was 1.4%, while the figure derived from urinalysis was 2.8% [26]. The similarity between the estimate derived from urinalysis and that of this study appear to support, though indirectly, the validity of capture-recapture method in estimating the prevalence of illegal drug use.

Cumulative evidence has shown that the incidence of HIV/AIDS and HVC has been escalating among the injection-drug users (mainly heroin), and it was estimated that from January 2005 to October 2005, about two thirds of newly reported HIV/AIDS affected cases were drug-injection users [34,35]. Preliminary research on illegal drug-using inmates conducted by our team indicated that the prevalence of HCV-infection was 30.1% and 78.2% for illicit drug-, heroin- injection-drug- user (IDU) respectively [35]. Meanwhile, the prevalence of HIV-infection was 7.7% and 25.9% for illicit drug- and heroin-injection-drug- user (IDU) respectively. As pointed out by the growing number of heroin users in the first years of 21^st ^century in our analyses, Taiwan must urgently address the issues of heroin use and associated medical problems.

### Methodological consideration

In the present study, we have assumed that the arrested illegal drug users, including some severe cases that might have met the criteria of substance dependence or associated medical complications, were a random sample drawn from the population of illegal drug users in the community. This assumption may hold true given several facts and policies related to illegal drugs use in Taiwan. First, the use of heroin and methamphetamine is currently considered criminal and is subject to be arrested by law enforcers. Second, the adjudication for detoxification in the prison system only applies for those drug users evidenced by positive urinalysis, but not for those non drug-using arrestees who either smuggled or possessed any illegal drugs. This policy makes the subjects recruited from the detoxification units in judiciary system more homogenous in the sense that all subjects were actual illegal drug users. Third, the chance for illegal drug users to be arrested by the policeman seems independent on subjects' demographic background of interests (e.g., local residence). Thus, to some extent the process might be equivalent to the process of random

Another issue encountered as applying the capture-recapture method on the illegal drug use involves the assignment of interchanging or polydrug users. If coded as a user of single type of illegal drug in either system, those interchanging drug users would lead to a significant loss of overlapping size in the analysis of any single drug. Whereas the overlapping size is the main denominator on estimating undetectable size and its standard deviation, the loss of overlap will inflate the estimate and result in estimation bias, particularly with sparse size [36].

Several previous studies adopting the capture-recapture model to estimating the prevalence of illegal drug abuse utilized datasets of more than two sources, such as the judicial or police system, emergency room, forensic system, drug treatment service, or counseling programs [13-15]. The mutual independence assumption could be relaxed and the inter-system correlations could be, modeled by using multiple sources. However, the heterogeneity between these systems might be a concern. Strict case definition considering the homogeneity between multiple sources was suggested while using capture-recapture by more than 2 lists. Since heroin is one of illegal drugs with highest dependence liability, problematic users of heroin identified from different sources should be similar or comparable, and any extra data source should be considered in order to obtain valid estimation. However, it may not be the case for the estimates of methamphetamine users, given that the subjects, identified from forensic mortality system, the emergency room, or the drug treatment service, may indeed reflect a subgroup characterized with more severe forms of substance use disorders than those identified from the judicial system. It is possible that the existence of such heterogeneity between different systems may violate a basic assumption for multiple-systems capture-recapture approach and therefore result in biased estimates [9-12].

## Conclusion

On the basis of the approach of capture-recapture method, our results found an increasing trend for heroin use among males in Taoyuan County, Taiwan. In contrast, the population of methamphetamine users seems downsizing from 1999 to 2002. These findings provide public health and clinical practice important information that may guide the planning and implementation of measures for future evidence-based intervention/prevention strategies and healthcare delivery.

## Competing interests

The author(s) declare that they have no competing interests.

## Authors' contributions

SCC was the principal investigator, contributed to study design, data collection, and statistical analyses. CYC contributed to the interpretation and writing of the article, and YYC and HJS contributed in the collection of hospital cases and the interpretation of the findings. WJC participated in study design, data analysis, and the interpretation of the findings and the editing of the article. All authors read and approved the final manuscript.

## Pre-publication history

The pre-publication history for this paper can be accessed here:


